# Smartwatch Electrocardiograms for Automated and Manual Diagnosis of Atrial Fibrillation: A Comparative Analysis of Three Models

**DOI:** 10.3389/fcvm.2022.836375

**Published:** 2022-02-04

**Authors:** Saer Abu-Alrub, Marc Strik, F. Daniel Ramirez, Nadir Moussaoui, Hugo Pierre Racine, Hugo Marchand, Samuel Buliard, Michel Haïssaguerre, Sylvain Ploux, Pierre Bordachar

**Affiliations:** ^1^Cardio-Thoracic Unit, Bordeaux University Hospital (CHU), Bordeaux, France; ^2^IHU Liryc, Electrophysiology and Heart Modeling Institute, Fondation Bordeaux Université, Bordeaux, France; ^3^Cardiology Department, CHU Clermont-Ferrand, Clermont-Ferrand, France; ^4^Division of Cardiology, University of Ottawa Heart Institute, Ottawa, ON, Canada

**Keywords:** electrocardiogram, atrial fibrillation, wearable, arrhythmia, diagnosis

## Abstract

**Aims:**

The diagnostic accuracy of proprietary smartwatch algorithms and the interpretability of smartwatch ECG tracings may differ between available models. We compared the diagnostic potential for detecting atrial fibrillation (AF) of three commercially available smartwatches.

**Methods:**

We performed a prospective, non-randomized, and adjudicator-blinded clinical study of 100 patients in AF and 100 patients in sinus rhythm, patients with atrial flutter were excluded. All patients underwent 4 ECG recordings: a conventional 12-lead ECG, Apple Watch Series 5®, Samsung Galaxy Watch Active 3®, and Withings Move ECG® in random order. All smartwatch ECGs were analyzed using their respective automated proprietary software and by clinical experts who also graded the quality of the tracings.

**Results:**

The accuracy of automated AF diagnoses by Apple and Samsung outperformed that of Withings, which was attributable to a higher proportion of inconclusive ECGs with the latter (sensitivity/specificity: 87%/86% and 88%/81% vs. 78%/80%, respectively, *p* < 0.05). Expert interpretation was more accurate for Withings and Apple than for Samsung (sensitivity/specificity: 96%/86% and 94%/84% vs. 86%/76%, *p* < 0.05), driven by the high proportion of uninterpretable tracings with the latter (2 and 4% vs. 15%, *p* < 0.05).

**Conclusion:**

Diagnosing AF is possible using various smartwatch models. However, the diagnostic accuracy of their automated interpretations varies between models as does the quality of ECG tracings recorded for manual interpretation.

## Introduction

Atrial fibrillation (AF) is the most common sustained arrhythmia in clinical practice but often remains undiagnosed. The ability to record an ECG tracing that is equivalent to lead I at any time and as often as desired is a relatively new feature of select smartwatches, creating opportunities to diagnose cardiac abnormalities such as AF (1–4). Recent guidelines recognize the potential value of smartwatch-based ECGs for diagnosing AF (5). Apple, Inc (Cupertino, CA, USA) released the first smartwatch to receive FDA approval for automated detection of AF, but smartwatches from competitors such as Samsung (Seoul, South Korea) and Withings (Issy les Moulineaux, France) can similarly record ECG tracings and warn wearers when AF is detected (6). The process of recording an ECG, analyzing it to generate an automated diagnosis of AF, and providing options to transmit these results to the wearer's physician(s) are similar between smartwatch manufacturers. However, their diagnostic algorithms are proprietary and not made available for analysis. The diagnostic accuracy of these algorithms and the ability of healthcare professionals to correctly interpret smartwatch-based ECGs may differ between commercially available smartwatches. Given this technology's widespread and growing use, mass screening for AF using various smartwatch-based technologies may effectively soon occur, the results of which will require clinical decisions on the part of healthcare professionals. Critical evaluation of the relative diagnostic strengths and weaknesses of commercially-available smartwatch technologies is therefore critical. The primary objective of our study was to compare the diagnostic performance of smartwatch ECGs from three companies (Apple, Samsung, and Withings), specifically their ability to accurately differentiate sinus rhythm (SR) from AF using either their automated algorithms or through review of recorded smartwatch ECG tracings.

## Methods

This was a prospective, non-randomized, and blinded clinical study of 100 consecutive patients in sinus rhythm who had undergone an AF ablation procedure in the previous 6 months and 100 consecutive patients in persistent or permanent AF who were referred for catheter ablation. All patients were ≥18 years of age and provided informed consent. Patients with atrial flutter, permanent pacemakers or implantable cardioverter-defibrillators were excluded. All patients had 12-lead ECGs performed, which served as the reference standard for the diagnosis of AF or sinus rhythm. Immediately after the 12-lead ECG was performed, 30-s ECG tracings using an Apple Watch Series 5® (Apple Inc, Cupertino, CA, USA), Samsung Galaxy Watch Active 3® (Samsung, Seoul, South Korea), and Withings Move ECG® (Withings, Issy-les-Moulineaux, France) were recorded in random order and after providing standardized instructions. These smartwatches' automated AF-detection algorithms yield one of several possible results, including “sinus rhythm,” “atrial fibrillation,” “low heart rate,” “high heart rate,” “poor recording” or “inconclusive recording.” All smartwatch ECG recordings were saved as PDF documents for offline analysis, anonymized, randomized and each automatic diagnosis was removed before distribution to two blinded electrophysiologists who independently interpreted each tracing and assigned one of three possible diagnoses: AF, SR, or unclassified (unable to differentiate between AF and SR). In addition, the quality of smartwatch ECG tracings was classified as good, poor but interpretable (e.g., presence of artifacts but differentiating between AF and SR was deemed possible), and uninterpretable. In case of disagreements between the two experts, a third cardiac electrophysiologist reviewed the tracing and made the final diagnosis.

### Statistical Analysis

For each of the three smartwatch models, sensitivity, specificity, positive predictive values and negative predictive values were calculated for automated and physician-interpreted smartwatch ECGs. Classifications were not binary as ECGs could be non-classified (i.e., inconclusive automated diagnoses or uninterpretable ECG tracings as per reading physicians) therefore two analyses were undertaken. In the first analysis, unclassified ECGs were considered false positives (when the patient was in SR) or false negatives (when the patient was in AF), yielding “worst-case-scenario” estimates (7). In the second approach, unclassified ECGs were excluded from the analysis. Kappa (κ) coefficients for interobserver agreement were assessed for the three models. Analysis of variance (ANOVA) tests were used to compare percentages between the three groups. All analyses were performed using SPSS software ver. 22.0 (IBM, Armonk, NY, USA) with a two-tailed alpha level of 0.05 to define statistical significance.

## Results

**Figure 1:**
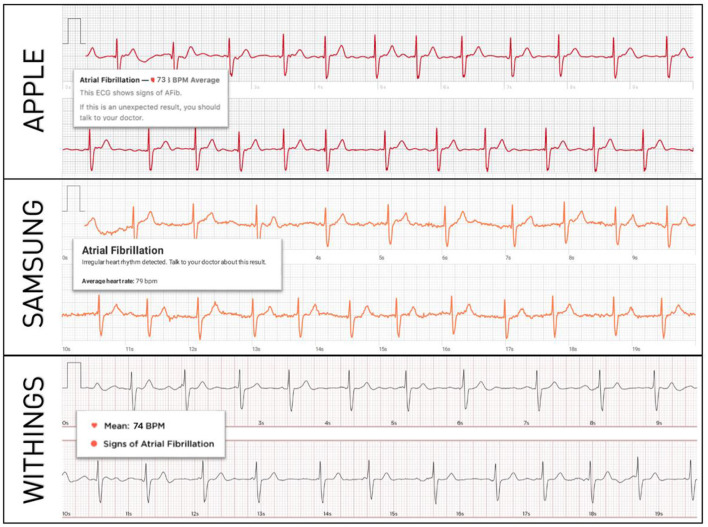
Representative examples of smartwatch ECGs in the same patient with confirmed AF. The diagnosis of AF is correctly made by each smartwatch's automated algorithm.

In total, 200 patients were enrolled (100 in SR, 100 in AF). Their mean age was 62 ± 7 years and 56% were male. Standard 12-lead and smartwatch ECGs from all the three models could be recorded in all patients, generating 200 12-lead ECGs and 600 single-lead smartwatch ECGs available for analysis. Representative examples of smartwatch ECGs from each model in a patient in AF is shown in Figure 1.

### Automated Diagnosis Using the Apple Smartwatch

Of the 100 patients in SR, 86 ECG recordings were correctly diagnosed as SR, 1 incorrectly as AF, and 13 were not classified (3 due to poor recording, 3 due to a heart rate of <50 beats/min, and 7 due to inconclusive recordings). Of the 100 patients in AF, 87 ECG recordings were correctly diagnosed as AF, 7 incorrectly as SR, and 6 were not classified (1 due to poor recording, 1 due to a heart rate of <50 beats/min, 1 due to a heart rate of >150 beats/min, and 3 due to inconclusive recording). When considering non-classified ECGs as false results, sensitivity was 87% (95%-CI 79–93%) and specificity 86% (95%-CI 78–92), positive predictive value (PPV) was 86% and negative predictive value (NPV) was 87 %. When excluding unclassified ECGs from the analysis, sensitivity was 99% (95%-CI 94–100%) and specificity 93% (95%-CI 85–97%), PPV was 93% and NPV was 99%.

### Automated Diagnosis Using the Samsung Smartwatch

Of the 100 patients in SR, 81 ECG recordings were correctly diagnosed as SR, 6 incorrectly as AF, and 13 were not classified (2 due to poor recording, 1 due to a heart rate of <50 beats/min, and 10 due to inconclusive recordings). Of the 100 patients in AF, 88 ECG recordings were correctly diagnosed as AF, 5 incorrectly as SR, and 7 were not classified (all 7 were considered inconclusive). When considering unclassified ECGs as false results, sensitivity was 88% (95%-CI 80–94%) and specificity 81% (95%-CI 72–88%), PPV was 82% and NPV was 87%. When excluding unclassified ECGs, sensitivity was 94% (95%-CI 87–98%) and specificity 94% (95%-CI 87–98%), PPV was 95% and NPV was 93%.

### Automated Diagnosis Using the Withings Smartwatch

Of the 100 patients in SR, 80 ECG recordings were correctly diagnosed as SR, 3 incorrectly as AF, and 17 were not classified (1 due to poor recording, 3 due to a heart rate of <50 beats/min, 1 due to a heart rate >100 beats/min, and 12 due to inconclusive recordings). Of the 100 patients in AF, 78 ECG recordings were correctly diagnosed as AF, 2 incorrectly as SR, and 20 were not classified (all were labeled as inconclusive). When considering non-classified ECGs as false results, sensitivity was 78% (95%-CI 68–86%) and specificity 80% (95%-CI 71–87%), PPV was 80% and NPV was 78%. When excluding non-classified ECGs, sensitivity was 96% (95%-CI 90–99%) and specificity 98% (95%-CI 92–100%), PPV was 98% and NPV was 96%.

### Comparison Across Smartwatch Models

**Figure 2:**
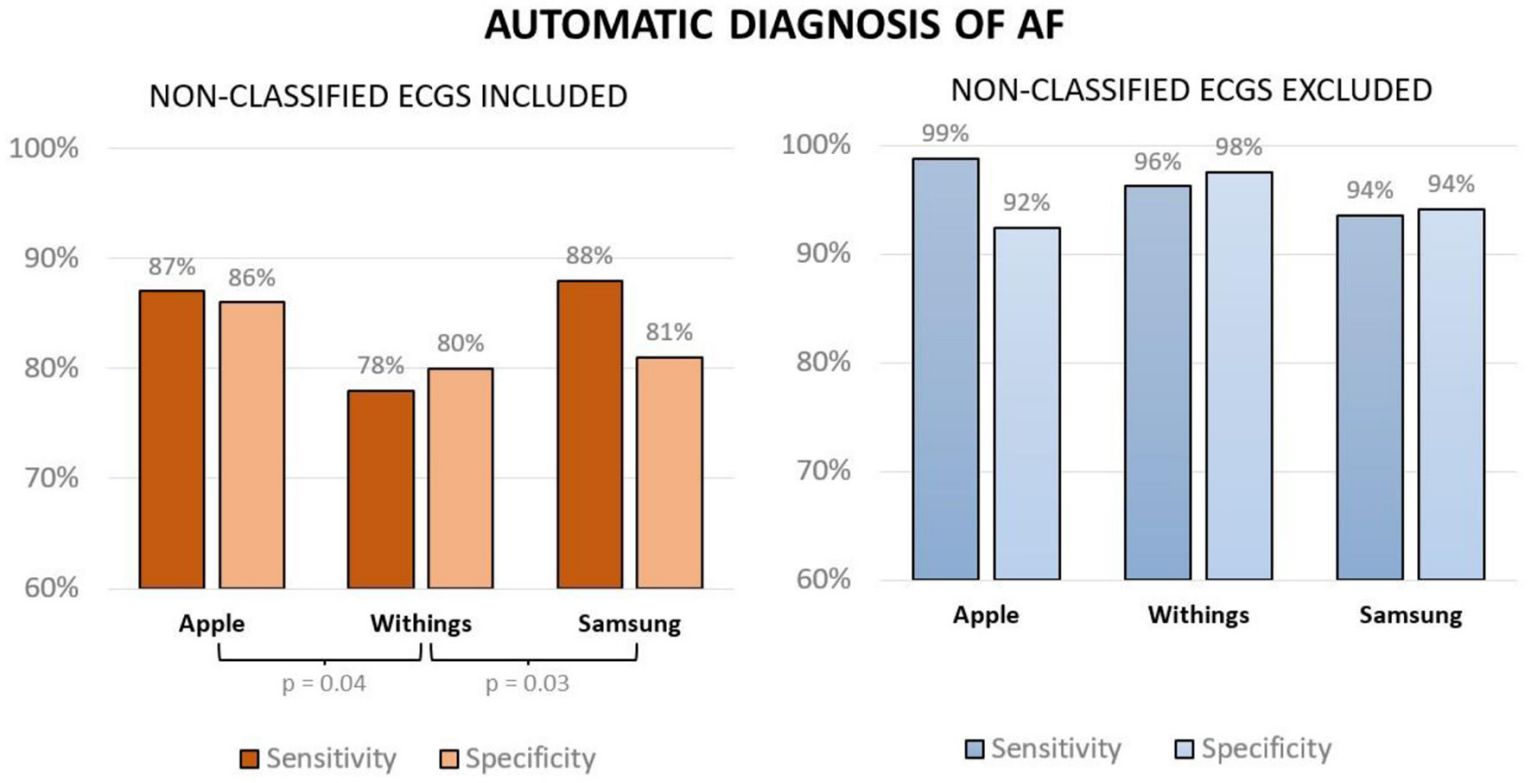
Sensitivity and specificity of smartwatch-based automated diagnoses of AF when considering unclassified ECGs as false results (left panel) and when excluding unclassified ECGs (right panel).

We presented the results separately for SR and AF since inconclusive diagnoses may differ between rhythms. All automated smartwatch algorithms had high sensitivity and specificity for the diagnosis of AF even when considering unclassified tracings as false results (Figure 2). However, the Withings smartwatch had lower sensitivity and specificity relative to Apple (*p* = 0.02 for comparison of sensitivity and specificity between Withings and Apple) and Samsung models (*p* = 0.03 compared with Withings) when unclassified ECGs were considered false results, possibly due to the higher proportion of unclassified ECGs with this smartwatch (19 vs. 10% and 10% respectively, *p* < 0.05).

### Manual Diagnosis by Electrophysiologists

**Figure 3:**
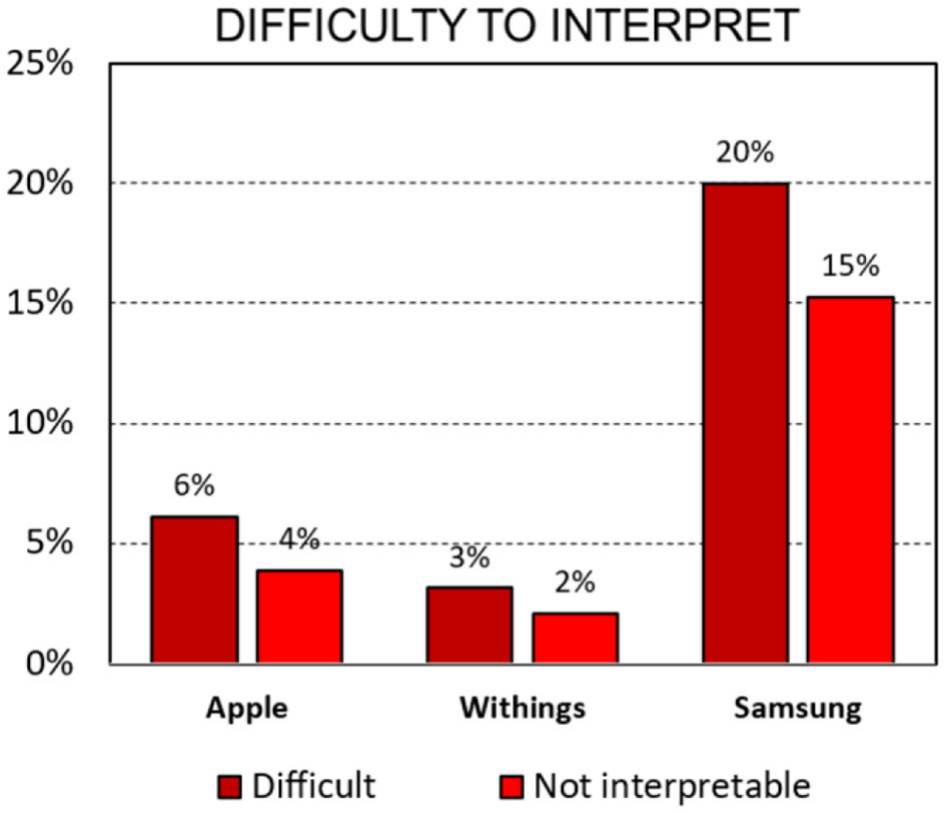
Percentage of *difficult* or *uninterpretable* smartwatch ECGs according to the experts (*p* < 0.05 for ANOVA analysis).

**Figure 4:**
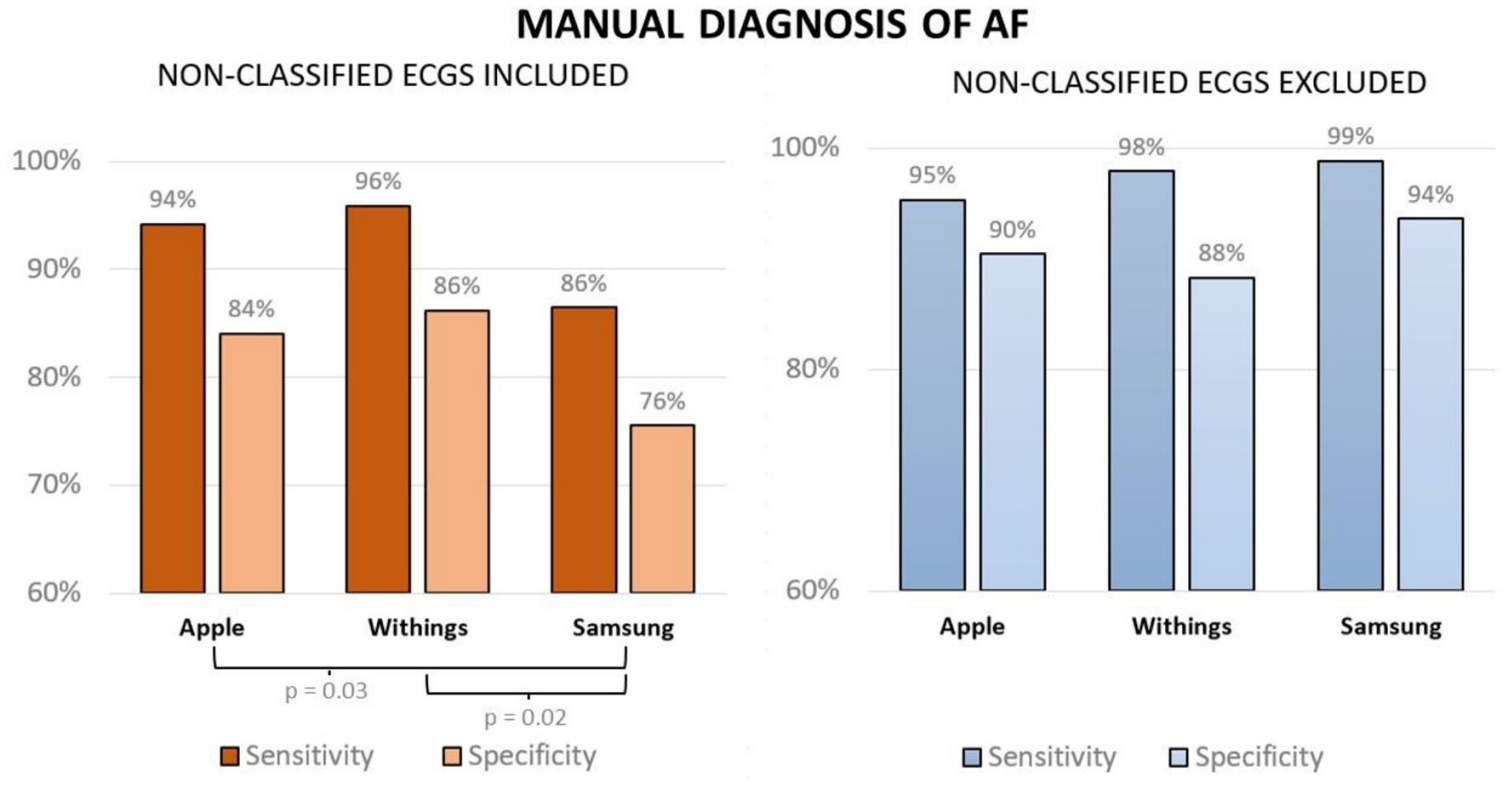
Sensitivity and specificity of expert-interpreted smartwatch ECGs for the diagnosis of AF when considering unclassified ECGs as false results (left panel) and when excluding unclassified ECGs (right panel).

Cardiac electrophysiologists exhibited high agreement for the differentiation between AF and SR with high inter-observer reproducibility for the three models (Apple κ= 0.96, Samsung κ= 0.92, Withings κ= 0.94). With 20% of tracings deemed difficult to interpret and 15% deemed uninterpretable, ECGs recorded with the Samsung smartwatch were more challenging for the electrophysiologists relative to the other models (Figure 3, Apple: 6% difficult and 4% uninterpretable; Withings: 3% difficult and 2% uninterpretable, ANOVA *p* < 0.05). When excluding uninterpretable ECGs, the sensitivity and specificity were high for all three models: 95% sensitivity and 90% specificity for Apple (PPV 90%, NPV 96%), 98% sensitivity and 88% specificity for Withings (PPV 89%, NPV 98%), and 99% sensitivity and 94% specificity for Samsung (PPV 93%, NPV 99%). When considering unclassified tracing as false results, the results were as follows: 94% sensitivity and 84% specificity for Apple (PPV 84%, NPV 94%), 96% sensitivity and 86% specificity for Withings (PPV 88%, NPV 95%), and 86% sensitivity and 76% specificity for Samsung (PPV 78%, NPV 85%) (Figure 4).

## Discussion

Direct access to wearable devices equipped with portable ECG technology is now widespread. This feature may prove useful for detecting symptomatic and asymptomatic AF, thus creating opportunities to intervene. Previous studies have always investigated a single model, mostly focusing on optical sensors and connected ECG wristbands (8–11). However, the relative diagnostic value of available smartwatch models is poorly known. Our results show that the accuracy of automated algorithms for the diagnosis of AF vary between smartwatch models as does the quality of ECG tracings recorded for offline interpretation by healthcare professionals.

### Automated Diagnoses: Sinus Rhythm vs. Atrial Fibrillation

Algorithm-based automated AF diagnoses may have undesired consequences. A less-than-perfect screening test used in a population with low pre-test probability of cardiac arrhythmias translates into a modest post-test probability of disease. False positives can be associated with anxiety, unnecessary medical testing, and even potentially inappropriate treatments. On the other hand, false negatives (diagnoses of SR or inconclusive rhythm when the patient is in AF) can falsely reassure the patient and lead to diagnostic and therapeutic delay. The results of our study show that the sensitivities and specificities of all three algorithms are high. While the Withings algorithm is associated with a slightly but significantly lower sensitivity, this may be due to the higher proportion of ECGs reported as inconclusive with this smartwatch. Inconclusive rhythm classifications may occur in several circumstances: if the heart rate is too high (depending on the model), the heart rate is too slow, the patient is in an arrhythmia other than AF, the tracing is of low quality and uninterpretable by the algorithm, or criteria are not met to classify the rhythm as SR or AF. The proportion of inconclusive tracings is expected to diminish as improvements in filtering, changes in algorithms, and widening of interpretable heart rate windows are implemented. For instance, the heart rate threshold above which AF is not diagnosed has been recently increased from 120 to 150 bpm in Apple smartwatches. The impact of inconclusive recordings may also be reduced with more patient practice, repeated recordings over time and alternative smartwatch positions (12–14). Artificial intelligence approaches may also improve the accuracy of automated diagnoses of smartwatches (15, 16). Alternative over-the-counter technologies to self-diagnose AF have also shown excellent accuracy among which ECG devices (such as AliveCor® 6L) and photoplethysmography-based smartphone apps (such as FibriCheck®) (17, 18). Smartwatches are expected to be more often used then mentioned alternative technologies as they are mostly acquired for non-medical purposes, not motivated by a healthcare professional.

### Quality of the Tracings and Interpretation by Electrophysiologists

**Figure 5:**
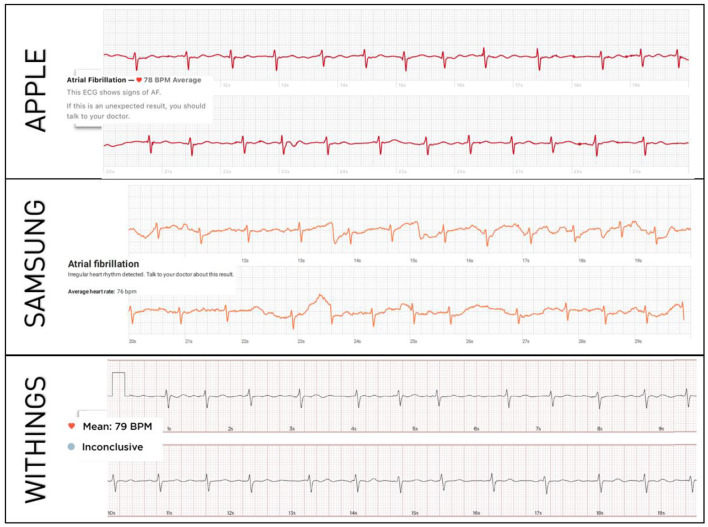
Smartwatch ECGs in the same patient with confirmed AF. Although the Samsung ECG was classified as difficult to interpret due to artifact, its automated algorithm correctly diagnosed AF. In contrast, the Withings ECG is of high quality but its automated algorithm failed to diagnose AF.

The product user manuals of the different smartwatches caution that the automated diagnosis (SR vs. AF) is provided only for information purposes and is not intended to replace the analysis of the tracing by a qualified health professional. Even though the accuracy of automated AF diagnoses is high, it remains imperative that a healthcare professional confirm the diagnosis before any therapeutic decision is made. The role for direct-to-consumer ECG tools in future guidelines will be defined by their feasibility and accuracy as shown in validation studies. Our study highlights that ECG tracing quality can differ between models with a direct impact on their diagnostic value. In our study, the quality of the tracings was lower using Samsung devices, which rendered ECG interpretation more difficult (the example shown in Figure 5 was classified as difficult to interpret). In fact, for this model, the automated diagnosis of AF outperformed offline ECG interpretation by experts. This may be due to differences in the criteria used to diagnose AF between smartwatch algorithms and physicians. For existing devices, automated AF diagnoses are schematically based on the exclusion of heart rates that are too fast or too slow (with different thresholds used across models), on the irregularity of QRS complexes, and the absence of repetitive patterns associated with extrasystoles. A perfectly stable rhythm will therefore usually be classified as sinus rhythm and an irregular rhythm as AF without a dedicated analysis of atrial activity. In contrast, although the above features are considered by electrophysiologists, direct analysis of atrial activity is considered an essential component of the diagnosis of AF—a criterion that generally requires an ECG tracing without excessive artifact or baseline wander for at least a few seconds. Without this confirmation, physicians may be reluctant to diagnose AF even if suspected.

### Study Limitations

This was a single-center study of 200 patients, half of whom had AF and half of whom had undergone atrial ablation. The accuracy of these devices in a larger population with or without cardiovascular risk factors or previous cardiac interventions remains to be shown. Participants were instructed on how to use the smartwatch prior to obtaining each recording and their ability to record each tracing was directly observed. The performance of the algorithms and the quality of the recorded tracings may be less accurate in an ambulatory setting without this instruction. However, none of the patients who participated in our study had previously used these smartwatches. While examiners were blinded to the concomitant automatic diagnosis and to the manual diagnosis of the smartwatch ECGs of the other models in the same patient, they were not blinded to the smartwatch model as each model features distinct characteristics on the ECG which make the manufacturer identifiable. More in-depth information about filters and algorithms would facilitate the comprehension of differences in performance between the smartwatch models but unfortunately this information is not made publically available by the manufacturers.

## Conclusion

Diagnosing AF is possible using various ECG smartwatch models. Our study demonstrates that there exist differences in the diagnostic accuracy of their automated algorithms and in the quality of ECG tracings recorded, the latter of which influences the ability of healthcare professionals to make a manual diagnosis of AF.

## Data Availability Statement

The raw data supporting the conclusions of this article will be made available by the authors, without undue reservation.

## Ethics Statement

The studies involving human participants were reviewed and approved by Bordeaux University Hospital. The patients/participants provided their written informed consent to participate in this study.

## Author Contributions

SA-A, NM, HM, and SB: collection of tracings and inclusion of patients. MS, FR, SP, and HR: writing of the article. PB and MH: concept and approval of the study. All authors contributed to the article and approved the submitted version.

## Funding

This work received financial support from the French Government as part of the Investments of the Future Program managed by the National Research Agency (ANR) (Grant Number ANR-10-IAHU-04).

## Conflict of Interest

The authors declare that the research was conducted in the absence of any commercial or financial relationships that could be construed as a potential conflict of interest.

## Publisher's Note

All claims expressed in this article are solely those of the authors and do not necessarily represent those of their affiliated organizations, or those of the publisher, the editors and the reviewers. Any product that may be evaluated in this article, or claim that may be made by its manufacturer, is not guaranteed or endorsed by the publisher.
